# Robust free-breathing SASHA T_1_ mapping using high-contrast image-based registration

**DOI:** 10.1186/1532-429X-18-S1-P28

**Published:** 2016-01-27

**Authors:** Kelvin Chow, Yang Yang, Michael Salerno

**Affiliations:** 1 grid.412587.d0000000419369932University of Virginia Health System, Charlottesville, VA USA; 2 grid.27755.32000000009136933XBiomedical Engineering, University of Virginia, Charlottesville, VA USA

## Background

Myocardial T_1_ correlates with fibrosis, but detection of sub-clinical disease requires accurate and precise T_1_ measurements, which are limited in breath-hold (BH) techniques. Navigator-gating (NAV) enables longer free-breathing (FB) acquisitions, but residual cardiac motion causes blurring without image registration. Variable flip angle (VFA) SASHA's [1] high accuracy and independent image acquisitions are well suited to this approach, however poor blood-tissue contrast makes accurate image registration difficult. We present a novel technique to generate high-contrast (HC) images to improve registration, enabling robust FB T_1_ mapping.

## Methods

**Figure 1:**
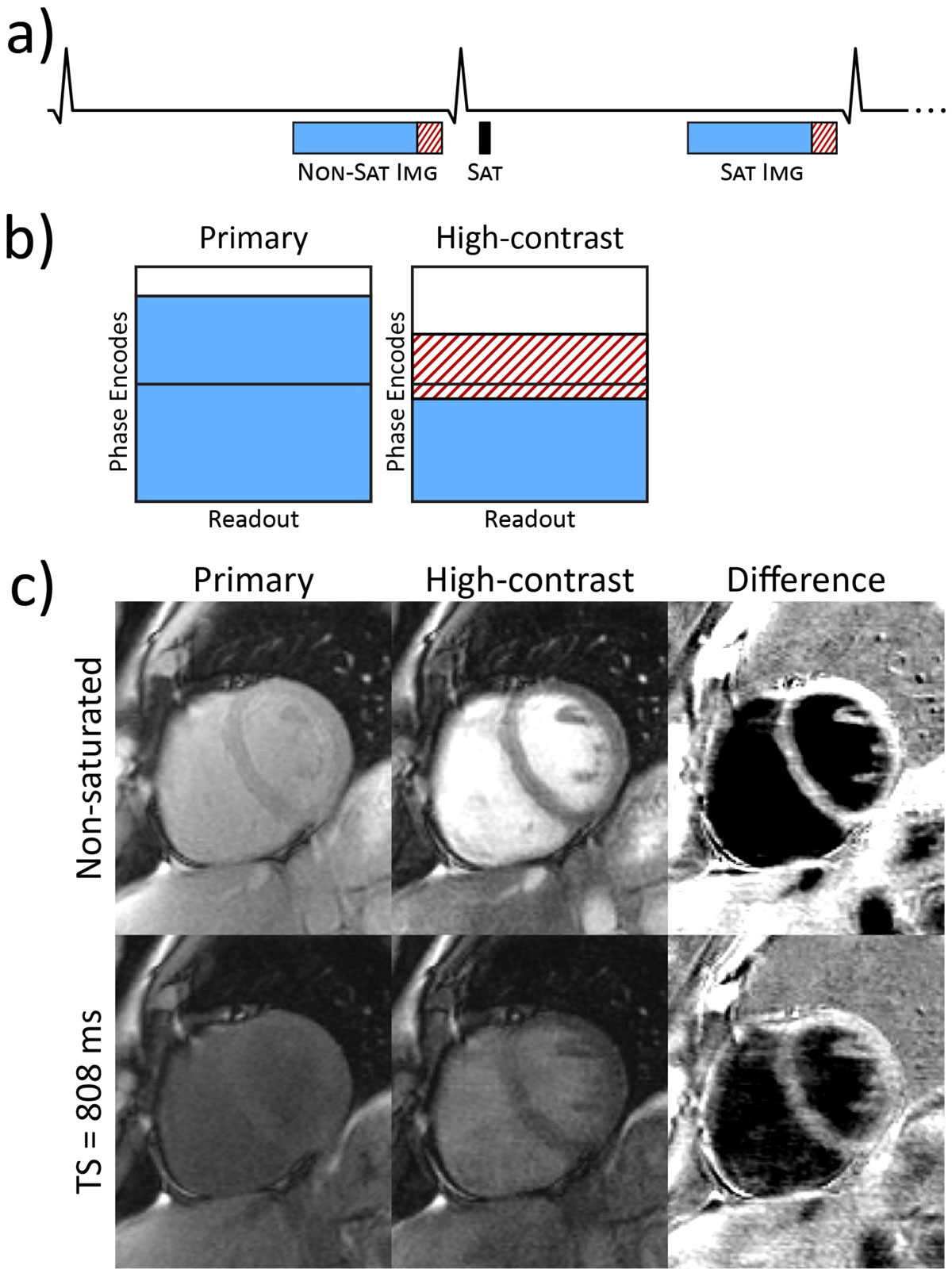
**a) Partial sequence diagram showing image acquisition for non-saturated and saturation recovery images, with primary image data marked in blue and high-contrast (HC) data in dashed red**. b) Acquired k-space for the primary SASHA-VFA images are reconstructed using GRAPPA and partial Fourier in the PE direction. The high-contrast image is created by replacing the center of k-space with the HC data in a key-hole fashion. c) Images from a healthy volunteer showing the primary SASHA-VFA images (used to calculate T1 maps), high-contrast images, and the difference between primary and high-contrast images for both non-saturated and TS images.

SASHA-VFA images have poor contrast due to the acquisition of the k-space center early in the bSSFP readouts. By increasing the flip angle to 120° after the k-space center, accumulated T_2_/T_1_ weighting generates blood-tissue contrast without affecting the accuracy of T_1_ maps calculated using the primary images. Additional low-frequency k-space lines collected following the primary images can be used to generate "key-hole" images with high contrast (Fig. 1), which can be used to motion correct the primary images. Sequence parameters include: 1.2/2.8 ms TE/TR, 340 × 255 mm FOV, 256 × 150 matrix, GRAPPA R=2, 65 phase encodes for the primary image, and 15 phase encodes at R=3 for the HC image.

14 healthy subjects were imaged on a 1.5T Siemens Avanto scanner. BH data was acquired with one non-saturated image and 10 TS images and FB data with 7-10 non-sat images separated by >5 seconds and 27-30 TS images for a total acquisition time of ~90 seconds. HC T_1_ maps were calculated using 50% of images selected automatically using an image based algorithm and registered using ANTs [2] with both the difference (Fig. 1) and primary images. NAV T_1_ maps were also calculated using images from these sets within a ± 3 mm window of a respiratory navigator. All T_1_ maps were calculated using a 2-parameter model, and the mean and coefficients of variation (COV) of myocardial T_1_ was determined. NAV and HC T_1_ maps were ranked by a blinded observer for myocardial border sharpness. Means and COVs were compared between the 3 techniques using repeated measures ANOVA with Tukey correction.

## Results

**Figure 2:**
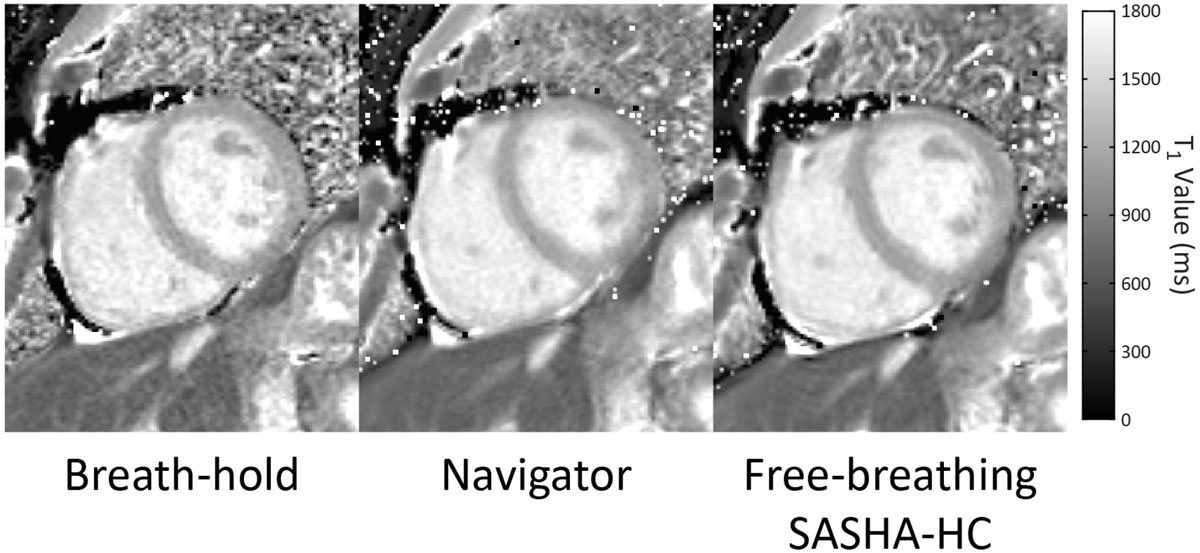
**T1 maps from a healthy volunteer using breath-hold (BH), navigator (NAV), and high-contrast (HC) with free-breathing image registration**. The BH map has lower precision than NAV or HC maps, and blurring of the septum, anterior left-ventricular endocardium, and papillary muscles are apparent in the NAV map as compared to HC.

T_1_ maps using BH, NAV, and HC are shown in Fig. 2. HC maps were ranked as sharper than NAV maps in 13 of 14 cases. Mean myocardial T_1_ with BH, NAV, and HC were 1152 ± 29 ms, 1159 ± 28 ms, and 1147 ± 28 ms respectively (p = NS). T_1_ COV was significantly lower for HC (4.2 ± 0.8%) compared with both NAV (4.9 ± 1.1%) or BH (5.9 ± 1.1%) (p < 0.05). T_1_ maps with BH, NAV, and HC used 11, 19 ± 6, and 22 ± 1 images respectively.

## Conclusions

A HC "key-hole" image can be acquired with <50 ms of additional data and used to improve image-registration. High-contrast SASHA is a robust approach to free-breathing T_1_ mapping, with HC T1 maps scoring sharper than navigator maps in 93% of cases and having 29% lower variability.
